# Auditory environmental context affects visual distance perception

**DOI:** 10.1038/s41598-017-06495-3

**Published:** 2017-08-03

**Authors:** Pablo E. Etchemendy, Ezequiel Abregú, Esteban R. Calcagno, Manuel C. Eguia, Nilda Vechiatti, Federico Iasi, Ramiro O. Vergara

**Affiliations:** 1 Laboratorio de Acústica y Percepción Sonora, Escuela Universitaria de Artes, CONICET, Universidad Nacional de Quilmes, B1876BXD Bernal, Buenos Aires Argentina; 2 Laboratorio de Acústica y Luminotecnia. Comisión de Investigaciones Científicas de la Provincia de Buenos Aires. Cno. Centenario e/505 y 508, M. B. Gonnet, Buenos Aires Argentina

## Abstract

In this article, we show that visual distance perception (VDP) is influenced by the auditory environmental context through reverberation-related cues. We performed two VDP experiments in two dark rooms with extremely different reverberation times: an anechoic chamber and a reverberant room. Subjects assigned to the reverberant room perceived the targets farther than subjects assigned to the anechoic chamber. Also, we found a positive correlation between the maximum perceived distance and the auditorily perceived room size. We next performed a second experiment in which the same subjects of Experiment 1 were interchanged between rooms. We found that subjects preserved the responses from the previous experiment provided they were compatible with the present perception of the environment; if not, perceived distance was biased towards the auditorily perceived boundaries of the room. Results of both experiments show that the auditory environment can influence VDP, presumably through reverberation cues related to the perception of room size.

## Introduction

Visual perception of egocentric distance to an object (visual distance perception or VDP) has been studied since Leonardo da Vinci’s times^1^ until nowadays, where it remains the subject of both numerous and various studies. VDP has been studied in both outdoors and indoors, under both field and laboratory conditions, and using both virtual and real environments^2–7^. Visual scenes potentially contain many different sources of information (both binocular and monocular) of distance such as: relative size, interposition, angular declination, perspective, motion parallax, binocular disparity and convergence, among others^8–15^. Cumulated evidence has shown that, in well-lit environments where multiple visual cues are available, VDP is quite accurate for targets up to 20 m away^16–19^. However, when visual cues are restricted (for instance, by reducing ambient illumination), perception becomes less accurate^20, 21^. This last result suggests that the information provided by the place where the subject and the target are located, which we call “environmental-context information”, can also influence the perception of objects and events presented within it.

In this line, recent studies have shown that, even in the presence of multiple visual cues, VDP can be influenced by the visual environmental context. It has been shown that VDP relies on the integration of local patches of ground information into a global surface reference frame^22, 23^. Also, VDP of familiar objects in natural settings is conditioned by the structure of the surrounding visual field^24–27^. For example, Lappin *et al*.^25^, showed that both the accuracy and the precision of distance judgments differed between three types of environments with multiple visual cues: a lobby, a hallway, and an open lawn. Furthermore, Witt *et al*.^26^ showed that, both in indoor and outdoor environments, the space beyond the target influences VDP. Finally, Stefanucci *et al*.^24^ reported that VDP is influenced by the inclination of the ground: participants perceived the distance to the targets at greater distances in steep terrain than in flat ground. These studies demonstrate that these sources of information serve as a complement for the classical VDP cues.

Another aspect that must be considered in space perception (and in particular in VDP) is calibration. The key point of calibration is that the several cues involved have different units related to internal variables of the body (i.e. embodied). In order to provide an accurate substrate to the actions performed by the subject^28^, two requirements need to be met. Embodied units must be accurately mapped to the units relevant for action, and also they must be accurately mapped to each other^29^. Several studies have focused on the rules governing calibration. For example, it has been shown that the relative stability of perceptual units determines which ones are used to recalibrate others^30^. Also, calibration of one action does not generalize to another action involving a different unit of action^31, 32^, but recalibration of perceptual units generalizes to the actions affected by those units^32^. Finally, it has been demonstrated that calibration transfers between limbs^31, 33^, but it also can be specified independently for each one, meaning that calibration is both functional and anatomical^34^.

It is interesting to analyze the influence of the environmental context on VDP within the framework of perceptual theories of sensory integration and sensory combination^35^. Sensory integration refers to the processing of information about the same (redundant) aspect of some environmental property: in VDP, the various distance cues provided by the visual target. On the other hand, sensory combination refers to the processing of information regarding complementary (non-redundant) aspects of the observed environmental property: in this case, information provided by the environment, the surrounding objects, and by prior knowledge, among others. These sources of information are collected aiming to reduce the inherent incompleteness of our perception of the world. Interestingly, both sensory integration and combination are also useful strategies for allowing cooperation between the different modalities, especially when a single modality is not enough to produce robust estimates. A renowned example of audio-visual integration is the “ventriloquist effect”, where the presence of a localized visual stimulus biases the localization in angle of a sound source^36–38^. This effect can be explained by a simple model of optimal integration of visual and auditory spatial cues, where each modality is weighted by an inverse estimate of its variability^39^. This kind of integration has also been demonstrated for visual-proprioceptive^40^, visual-tactile^41^ and visual-haptic^42^ interactions.

With regard to multimodal sensory combination, recent studies have shown that the context information can affect auditory distance perception in a multimodal fashion. For instance, a study carried out by Calcagno *et al*.^43^ showed that visual range information about the whole scene increases the accuracy of auditory distance judgments even when the sound source itself is not visible. The results of this study also show that auditory distance perception is significantly improved if participants are allowed to observe the experimental room minutes before performing the experiment in complete darkness. The authors hypothesized that the perceived distance of the sound source was calibrated by information related to the size of the experimental room. This hypothesis is in line with the proposal recently made by Gajewsky *et al*.^44, 45^. These authors postulated that the representation of the surrounding space can serve as a structured spatial reference into which distance cues are integrated. As a consequence, the reference frame provided by the environment can contribute to the scale of perceived distance within it, constraining or expanding the response. In a recent paper, Kolarik *et al*.^46^ tested this hypothesis and observed a positive correlation between auditory distance perception and auditory room size perception. Their results strongly suggest that also the auditory environmental context influences the auditory perception of distance.

Auditory context information (mainly associated to reverberation cues) has proven useful for the perception of the surrounding space. Human subjects can correctly match photographs of rooms with the corresponding binaural recordings^47^. Also, blindfolded subjects can distinguish the size of a room by using speech and other reflected sounds^48^. Furthermore, several researchers have found a direct relation between reverberation and perceived room size: greater reverberation time is consistently associated with larger rooms^46, 49–51^. In summary, although we may not be aware of it, in everyday experience humans employ a great deal of auditory information in order to perceive the surrounding environment, complementing the information from other senses.

Given this evidence, we wonder whether the perceived distance to a visual object can be influenced by environmental-context information coming from the auditory modality. For example, if as described earlier, visual information from the environment affect the perception of distance to an auditory object, could the reciprocal be true? In other words, is it possible, under certain conditions, to obtain a representation of the environment through auditory information, which in turn influences VDP?

Here, we investigated whether auditory environmental context influences the perceived distance to luminous objects located in two dark rooms with extremely different reverberation times: an anechoic chamber and a reverberant room. We hypothesize that visual distance perception could be biased towards an estimate of the size of the surrounding space obtained mainly through auditory cues.

## Results

### Experiment 1

**Figure 1:**
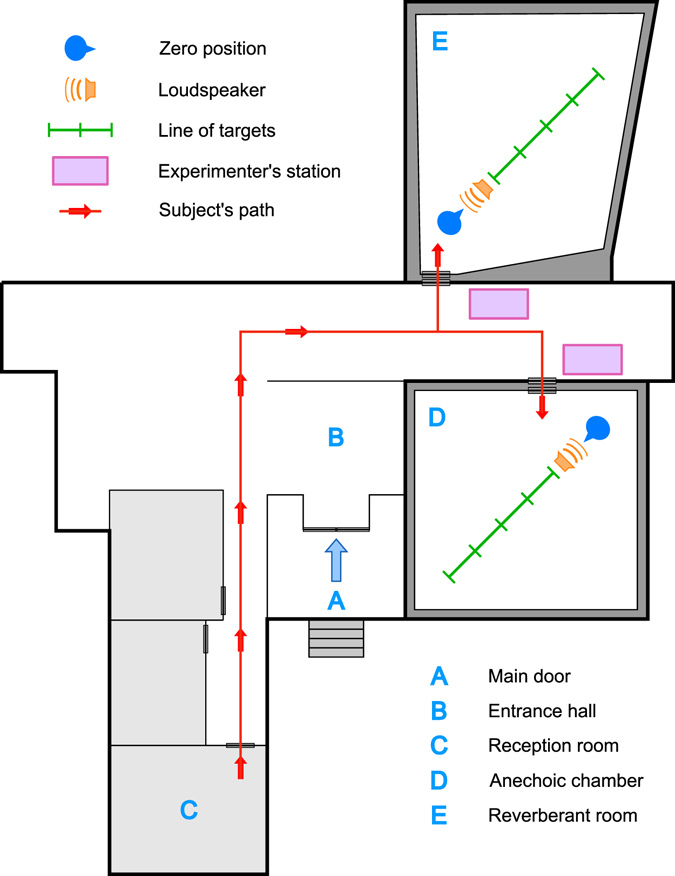
Schematic diagram of the experimental set-up at the Laboratorio de Acústica y Luminotecnia: It can be observed the reverberant room (E), the anechoic chamber (D), the path walked by the subjects (with eyes covered) during the experiments since they were received (B) to the reception room (C, where they were debriefed) and to both testing rooms (red line and arrows). Also shown are the experimental stations and the layout of the experimental set-up in both rooms.

Subjects where randomly assigned to one of two rooms among an anechoic chamber (Group 1, *n* = 37, mean reverberation time *T* = 0.12 s) and a reverberant room (Group 2, *n* = 38, mean *T* = 3.9 s; see Supplemental Table S1). None of them had prior knowledge of any of the two experimental rooms or their dimensions. Subjects were presented with luminous square targets of size 5 × 5 cm, located at distances *D* = 2, 3, 4, 5 and 6 m measured from the subject’s seat. Target’s luminance was adjusted to prevent any surface of the room from being illuminated. In this way we ensured that during the task participants could only see the experimental targets. In each trial, subjects had to report verbally the perceived distance to the target. The instructions, reproduced through a loudspeaker, as well as the verbal reports of the participants, were some of the auditory stimuli used to expose them to the acoustic characteristics of each room. See Methods and Fig. 1 for details on the procedure.

**Figure 2:**
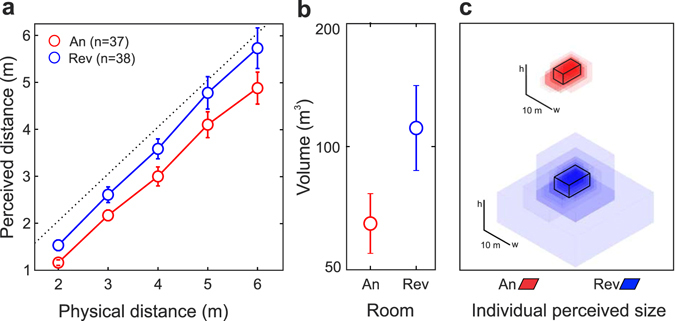
Results of Experiment 1: (**a**) Visual distance perception curves for both rooms in Experiment 1. We show mean responses (±SEM) as a function of target distance: anechoic chamber (An) in red and reverberant room (Rev) in blue. The black dotted line indicates perfect performance. (**b**) Mean perceived volume (±SEM) for each room. (**c**) Each transparent box corresponds to the individual perceived dimensions (width, length and height) for each condition (anechoic chamber: upper section, in red; reverberant room: lower section, in blue). For each room, the box in solid black lines represents the average of each dimension (taken separately) across subjects.

Figure 2a presents the average perceived distance (±SEM) as a function of the target distance for both groups. In both rooms the response was almost linear and the slopes were fairly similar (anechoic chamber: 0.94 ± 0.08; reverberant room: 1.06 ± 0.10). Also, both groups underestimated the distance to the target for all testing distances. Interestingly, participants who performed the experiment in the reverberant room (Group 2) systematically perceived the visual targets at greater distances than participants assigned to the anechoic chamber (Group 1). For instance, targets located at 5 and 6 m differed in 68 and 85 cm respectively, resulting in a relative difference of roughly 17%. The statistical significance of the difference was assessed by means of a repeated-measures ANOVA on the logarithms of the responses, which revealed a significant effect of the room [*F*(1,73) = 4.11, *p* = 0.046] and of the target distance [*F*(4,292) = 627, *p* < 0.001] but not of their interaction [*F*(4,292) = 0.97, *p* = 0.37].

In order to test whether a relationship between VDP and room size perception exists, after completion of the VDP task we asked the participants to estimate the room size (see Methods). Results are plotted in Fig. 2b (mean perceived room volume) and Fig. 2c (mean and individual perceived length, width and height). The reverberant room was perceived significantly larger than the anechoic chamber [*t*(73) = 1.83, *p* = 0.035, mean ratio = 1.71]. In order to explore this association, we also analyzed the correlation between the perceived room size and the maximum perceived distance (MPD) in log-log scale (see ref. 46). We obtained a significant positive correlation between MPD and the perceived volume in both rooms (anechoic chamber: *r* = 0.48, *p* = 0.0029; reverberant room: *r* = 0.65, *p* = 1.2 × 10^−5^; see Supplemental Figure S2).

It is interesting to analyze our results from the point of view of calibration^29^. If the calibration reference (in our case, the perceived room size) is modified by a certain multiplicative factor without recalibration, it should be expected that the perceived distance be modified by the same factor. As a consequence, the slope of the linear function relating target distance with perceived distance should change^30^. Here, the reference provided by the auditory environment was different for each room; therefore, the slope of the VDP response should be different in each case. We tested this prediction by applying linear fits to each subjects’ data and comparing differences in the parameters across rooms. We found that neither the slope nor the ordinate were significantly different across rooms [slope: *t*(73) = 0.94, *p* = 0.35; intercept: *t*(73) = 0.38, *p* = 0.71]. One possibility is that the lack of significance of this test could be due to the high variability of our data compared to previous studies on calibration. That said, we analyzed the correlation between the individual perceived room size (in log scale) and the slope of the response for each experimental condition (see Supplemental Figure S3). If the prediction of the calibration theory holds, a larger slope should be associated with a larger perceived room size. As expected, both magnitudes were positively correlated for both groups (Group 1: *r* = 0.35, *p* = 0.032; Group 2: *r* = 0.39, *p* = 0.017). This analysis suggests that the perceived room size affected the calibration of the VDP response.

### Experiment 2

We performed a second experiment to study whether the auditory contrast given by the consecutive exposure to two rooms with extremely different auditory characteristics has some effect on the perception of the room size and, therefore, on the perceived distance of the visual targets. To this end, subjects from Group 1 repeated the VDP task but in the reverberant room (i.e., they went from the anechoic chamber to the reverberant room, which will be referred to as A → R) while subjects from Group 2 repeated the VDP task in the anechoic chamber (i.e., they went from the reverberant room to the anechoic chamber, referred to as R → A). Experiment 2 was performed few minutes after completion of Experiment 1, following exactly the same procedure as in the first room (see Methods), and with the same subjects. In particular, the instructions of the task were repeated through the loudspeaker, just as before in Experiment 1. At this point, subjects were able to experience the strong auditory contrast between both rooms due to their extremely different reverberation times. Responses were also made verbally, contributing as well to the auditory experience.

**Figure 3:**
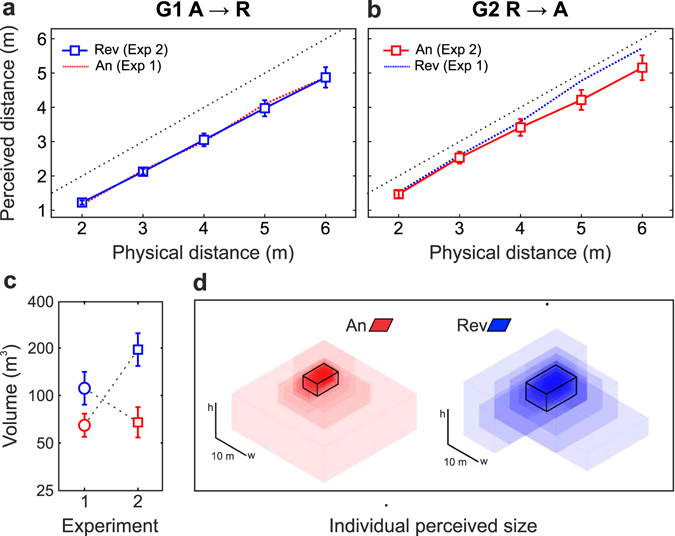
Results of Experiment 2 and comparison with Experiment 1: (**a**) Distance curves (mean response ± SEM as a function of target distance) for Group 1. Subjects went from the anechoic chamber (Experiment 1, red dotted line) to the reverberant room (Experiment 2, blue solid line). (**b**) Same as previous for Group 2. Subjects went from the reverberant room (Experiment 1, blue dotted line) to the anechoic chamber (Experiment 2, red solid line). In both panels, the black dotted lines indicate perfect performance. (**c**) Mean perceived volume for each room in both experiments. Data for each group are joined with dotted lines. (**d**) Each transparent box corresponds to the individual perceived dimensions (width, length and height) for each condition (anechoic chamber: left section, in red; reverberant room: right section, in blue). For each room, the box in solid black lines represents the average of each dimension (taken separately) across subjects.

In a manner similar to Experiment 1, we observed a significant effect of the room on perceived room size (Fig. 3c: mean perceived room volume, and Fig. 3d: mean and individual perceived length, width and height). Independently of the order of exposure, subjects within each group perceived the reverberant room larger than the anechoic chamber [Group 1, A → R: *t*(36) = 5.47, *p* = 1.8 × 10^−6^, mean ratio = 3.02; Group 2, R → A: *t*(37) = 3.85, *p* = 2.3 × 10^−4^, mean ratio = 1.64]. Moreover, comparison between groups showed that, in the second exposure, the reverberant room was perceived to be larger than the anechoic chamber [*t*(73) = 3.26, *p* = 8.4 × 10^−4^, mean ratio = 2.89]. Interestingly, the difference was larger than that reported in Experiment 1 (mean ratio = 1.71), suggesting that previous exposure to a room with opposite acoustical characteristics influences the perception of room size.

We next proceeded to analyze if perceived room size had any influence on VDP. Figure 3 shows average perceived distance (±SEM) as a function of target distance for Group 1 (A → R, Fig. 3a) and 2 (R → A, Fig. 3b). We compared the response of each group with the responses obtained by themselves in Experiment 1 by means of repeated-measures analysis of variance. Contrary to Experiment 1, the room showed a non-significant effect on VDP. Participants from each group perceived the targets at distances similar to those previously reported in Experiment 1 [Group 1: *F*(1,36) = 0.0273, *p* = 0.87; Group 2: *F*(1,37) = 4.06, *p* = 0.051]. Distance to the target was significant [Group 1: *F*(4,144) = 379, *p* < 0.001; Group 2: *F*(4,148) = 430, *p* < 0.001], while the interaction between room and target distance was not significant [Group 1: *F*(4,144) = 1.41, *p* = 0.24; Group 2: *F*(4,148) = 1.97, *p* = 0.12].

Although we did not observe a global difference in VDP responses in either group, we note that subjects from Group 2 (R → A) tended to compress their responses for the two farthest targets, which could be the cause for the marginal significance obtained in the ANOVA for the room (*p* = 0.051) for this group. We found that targets located at *D* = 5 and 6 m were perceived farther in the reverberant room than in the anechoic chamber [5 m: *t*(37) = 2.76, *p* = 0.0045 < 0.01, mean ratio = 1.12; 6 m: *t*(37) = 2.39, *p* = 0.011 < 0.02, mean ratio  = 1.10; both comparisons held their significance after applying the Holm-Bonferroni multiple-comparisons correction^52^, see Methods].

We next tested the association between MPD and perceived room volume. Interestingly, MPD positively correlated with perceived volume for Group 2 (R → A, anechoic chamber, *r* = 0.67 with *p* = 4.7 × 10^−6^) but did not correlate for Group 1 (A → R, reverberant room, *p* = 0.18; see Supplemental Figure S2). This last result is consistent with the presence (for Group 2) and absence (for Group 1) of an effect of the auditory environmental context on VDP, despite subjects from both groups were able to perceive differences in room size across rooms.

In order to study the calibration of the response with respect to the perceived room size, we obtained linear fits for each subjects’ data and tested for the existence of differences in the fit parameters across rooms. Neither the slope nor the ordinate were significantly different across rooms [slope: *t*(73) = 0.079, *p* = 0.94; intercept: *t*(73) = 1.58, *p* = 0.12]. As in Exp. 1, we also analyzed the correlation between the slope of the response and the perceived room size for each room (see Supplemental Figure S3). In a manner similar to the analysis of the correlation between MPD and perceived room size, we found a positive correlation for Group 2 (R → A, *r* = 0.64, *p* = 1.8 × 10^−5^) but not for Group 1 (A → R, *r* = 0.27, *p* = 0.11).

In order to account for these observations, we hypothesize that, once the subject has accustomed to the task (during Experiment 1), the change of the auditory environment (during Experiment 2) could be insufficient to change the distance perceived in the previous session, unless a contradiction exists between the previous response (especially over longer distances) and the present perception of the space. This hypothesis is able to account for the differential effect observed for subjects of Group 2 (R → A). These subjects went from the reverberant room to the anechoic chamber, and therefore experienced a reduction of the perceived space. It is possible that the farthest distances perceived in the reverberant room during Experiment 1 could lie beyond the perceived boundaries of the anechoic chamber during Experiment 2 and therefore they could not be possible (the average values for each dimension can be found in Supplemental Table S4). Subjects of Group 1, instead, experienced an expansion of the perceived space, and therefore all previous distance estimates were possible under their present perception of the room, thus remaining unchanged.

We tested this hypothesis by computing the percentage of subjects whose maximum perceived distance was greater than the reported length of the room (i.e., MPD ≥ Length﻿)﻿, which we will refer to as incompatibility index. Applying this definition to the data obtained for each group in each room, we obtained a baseline value for the incompatibility index. That is, the average proportion of subjects who provided at least one distance estimate lying outside the room. This resulted in a relatively low value (mean = 14.7% with 95% CI = ±3.4%, see Supplemental Table S5). We next applied the definition to the data of each group across both rooms: MPD (Exp. 1) ≥ Length (Exp. 2). For Group 1 (A → R) we obtained a value equal to 8.1%, the smaller value across conditions. These subjects maintained the VDP responses across rooms but reported an increase of perceived room size in Exp. 2 with respect to Exp. 1. Following our hypothesis, all distances perceived in Exp. 1 were possible in the larger room reported in Exp. 2. On the contrary, subjects of Group 2 (R → A) showed the largest value of all conditions (28.9%, see Supplemental Table S5). This value was also significantly different compared to the baseline value [*t*(3) = 8.18, *p* = 0.0038]. This implies that the maximum distances reported in Exp. 1 were larger than the room length perceived in Exp. 2, making them contradictory with the perceived room. This makes plausible that, during Exp. 2, subjects from Group 2 compressed their responses for the two farthest targets in order to make them fit with the auditorily perceived size of the new room. In fact, the incompatibility index for subjects of Group 2 in the anechoic chamber was 13.2%, indicating that they indeed adjusted their responses to the room size perceived under the new condition.

## Discussion

The experiments reported here demonstrated that the auditory environmental context affects the perceived distance of luminous targets located in the dark. In Experiment 1, participants placed in the reverberant room perceived all the targets farther than those placed in the anechoic chamber. We attribute the observed VDP differences to the acoustical differences between both rooms. Such assertion is based on the controlled conditions of the experiment: (i) all subjects had no prior knowledge of the room characteristics and they were located in the same relative position inside each room; (ii) testing distances and set-ups were identical in both rooms; and (iii) the rooms were located one in front of the other, thus the proprioceptive information that subjects could have acquired was also identical.

The effect of the auditory environment on VDP appears to be induced by differences in reverberation cues related to the auditory perception of the room size. Consistently, the subjects perceived the reverberant room of greater size than the anechoic chamber. This result agrees with many previous studies that reported a positive correlation between reverberation level and perceived room size^46–51^. The hypothesis about the relation between the perceived size of the room and the perception of distance has been proposed in both visual and auditory distance perception studies^43–46, 51^. Gajewsky *et al*.^44, 45^ proposed that the representation of the environment could contribute to the scale of perceived distance within the environment, constraining or expanding the response, especially for objects located near the perceived boundaries of the environment. In this line, Kolarik *et al*.^46^ found a positive correlation between auditory distance perception and auditory perception of room size through reverberation cues.

Following this reasoning, we argue that, under the conditions used in our study, the lack of prior knowledge of the room, combined with the complete absence of visual environmental cues, induced the participants to calibrate their response (perceived distance) to a room whose size could mainly be perceived through the auditory modality. First, this hypothesis is supported by the fact that the targets were perceived at a greater distance in the room that was perceived larger. Second, we found that the perceived distance of the most distant target (MPD) and perceived room volume were positively correlated.

According to the calibration literature^29, 30^, if the perceived room size calibrates the perceived distance, in the absence of feedback information the slope relating response with target distance should change between rooms. However, this was not observed. One cause could be the high variability of our data, compared to previous studies on calibration. First, both the accuracy and variability of the VDP responses are lower in a dark room compared to an illuminated room^21^. Second, perception of auditory space is, in general, more variable than perception of visual space^53, 54^. Moreover, perceived room size information does not change with target location, it only depends on the room in which the task is performed. Finally, unlike previous studies on calibration, in our study the association between reverberation level and room size was not established during a controlled phase of the experiment, but instead depended exclusively on the prior experience of the subjects. The combination of these factors could have obscured the effect of the calibration on the slopes. However, the fact that, in both conditions, the correlation between the slopes and the perceived room size was found positive and significant suggests that the perceived room size affected the calibration of the VDP responses.

In Experiment 2, subjects from both groups repeated the same VDP task but in the other test room. Based on the findings obtained in Experiment 1, we expected that subjects would report perceived distances accordingly to the reverberation level of each room, assigning farther distances as reverberation was increased. In fact, we expected an increased effect of the room on VDP due to the high auditory contrast experienced after changing rooms. However, this was not observed. When comparing results within each group, subjects did not systematically adjust their responses to the room’s reverberation level, despite they perceived significant differences in room volume when passing from one room to the other. Instead, subjects responded almost in the same fashion as they previously did in Experiment 1.

A possible explanation of this result is as follows. Even when the results of Experiment 1 show that the auditory context influenced the response, the perceived distance was modulated mainly by the visual cues provided by the targets. It is thus possible that, as subjects performed the task in the first room, a robust association developed between visual cues and distance estimates. Given that both the targets and the testing distances were identical in both experiments, and that in general visual cues are more reliable than auditory cues, it is possible that the change of the auditory context during the second exposure could be insufficient to change the previous association between visual cues and responses. This could have been favoured also by the prior familiarization with the stimulus (see Methods), and by the short time interval between experiments.

For this to happen, the participants had to keep in their memory these associations during the time between the end of Exp. 1 and the beginning of Exp. 2. Previous VDP studies have reported that visual information related to the distance to an object can be accurately stored in memory. For example, subjects can sight a target up to 20 m away or more, and then walk to it quite accurately while blindfolded^16, 18, 19, 53^. Also, Calcagno *et al*.^43^ showed that subjects can store in their memory a visual representation of the environment that improves, minutes later, the perception of distance to sound sources. These results suggest that it is possible that the visual information obtained in Experiment 1 affected the response during Experiment 2. One interesting question arising from this is how much time this information can be stored in memory before degrading. We do not know what results could have been obtained if Experiment 2 was performed a few hours, or a few days, after Experiment 1. Additional studies are needed to know the temporal course of this effect.

Interestingly, the only discrepances between Exp. 2’s and Exp. 1’s responses were observed in the responses of subjects from Group 2 (R → A). These subjects maintained their responses only for the nearest (2–4 m) but not for the farthest targets (5–6 m), which were perceived significantly closer in the anechoic chamber than in the reverberant room. Subjects from this group reported a reduction of the room size in the anechoic chamber (compared to that previously reported in the reverberant room in Experiment 1) enough to be incompatible with the farthest perceived distances previously reported in Experiment 1. This was clearly reflected by the incompatibility analysis between MPD and perceived room size, for which Group 2 displayed the larger value of the incompatibility index across conditions. Thus, we hypothesize that subjects preserved their responses from the previous VDP task provided they were compatible with the present perception of the surrounding space, i.e., targets should be perceived inside the perceived room; if not, VDP is biased towards the perceived boundaries of the room.

This hypothesis is supported by the analysis of correlation between MPD and the perceived volume of the room. Subjects from Group 2 adjusted the perceived distances in Experiment 1 (at least for the two farthest targets) to a room whose perceived size was smaller, and therefore they show a positive correlation coefficient between both variables. On the other hand, the absence of correlation for subjects from Group 1 summarizes the fact that they were able to perceive differences in size between both rooms without associating room size with the distances previously reported. The same result was observed in the correlation between the slope of the response and the perceived volume of the room. These results agree with our proposal that, during the second exposure, the participants only perceived changes in the distance of the target when it was incompatible with the perceived room size.

## Conclusions

The effect of the visual environmental context on VDP has been reported in several recent studies^22–27^. However, unlike previous studies, the results obtained here show that the interplay between the visual object and the environment can be formed in a multimodal way. Moreover, none of the previous studies explicitly considered the perceived size of the place in which the task was performed as an influential factor in VDP. The only similar antecedent is the study of Kolarik *et al*.^46^ where they observed a positive correlation between the distance of a source and the size of a room both perceived through the auditory modality. Although further study is needed to fully evaluate this relation, we consider that the results presented here strongly suggest that VDP depends on the perception of the size of the environment where the experiment is performed.

Multimodal auditory-visual interactions have been shown in many previous studies. When redundant information about some feature of an object comes from different senses (multisensory integration, see ref. 35), information is merged in such a way that a coherent multisensory percept is formed. Recent multimodal studies have shown that no modality has preponderance over others, but instead unimodal percepts are merged taking into account the quality of the information provided by each one. Under the conditions used here, environmental visual cues were degraded to the point that vision gave no information about the spatial context. In consequence, subjects had no other choice but to create a representation of the surroundings using other modalities, mainly auditory cues.

We consider that the effect reported here is moderate (of the order of 20%), especially considering that the experiment was conducted under extremely different reverberation conditions and in the absence of visual references from the environment. Presumably, if the experiment had been done in a normally lit room, the visual information would have overridden the effect of the auditory environment. However, our results argue that the information of the environmental context obtained through the auditory modality can create a spatial representation of the surroundings, and that such representation can serve as a reference frame for the location of objects perceived through a different sensory modality (in this case, visual). Previous studies of auditory distance perception have shown a similar relationship between the perception of the object and that of the environment, but in those cases the visual environment influenced the auditorily perceived distance of a source^43, 55^. Therefore, it is reasonable to expect a similar effect on distance perception of spatial information obtained from another sensory modality (e.g., proprioceptive).

The multimodal relationship observed here can serve as a model to better understand how the brain encodes spatial information from various senses. For example, it could help to guide experiments aiming to understand if brain maps are formed in a common area of the brain where all modalities contribute, or if, instead, each sensory modality forms a map in a zone of the brain specific for it. The idea of a multimodal environmental context in which each sensory modality contributes to construct our perception of the environment could also be used to improve the realism of virtual reality environments. Nonetheless, further research is needed to understand the extent and limitations of this kind of multimodal interactions, as well as the influence of other modalities on VDP.

## Methods

### Testing environments

The study was conducted in the Laboratorio de Acústica y Luminotecnia (LAL) of the Comisión de Investigaciones Científicas de la Provincia de Buenos Aires (CIC-BA). The LAL has two rooms with very different reverberation times (see Supplemental Table S1): (a) an anechoic chamber (mean *T* = 0.10 s) and (b) a reverberant room (mean *T* = 3.9 s). The anechoic chamber had a volume equal to 7.00 × 6.90 × 5.90 m (length × width × height) and a free working space equal to 5.40 × 5.30 × 4.30 m. The reverberant room was a seven-surface irregular polyhedron, approximately equivalent to a rectangular box 7 × 8 × 4 m in size, with a volume equal to 189 m^3^. For exact details of both chambers see refs. 56 and 57. Both rooms were located in the same hallway with their doors facing each other (see Fig. 1). Two identical experimental set-ups were mounted one in each room, located in the same relative position with respect to the door.

### Participants

A total of seventy-five volunteers (19 women and 56 men) participated in the experiments. None had prior knowledge of the experimental rooms or the set-up, nor were informed of any characteristic of the rooms. All subjects were naïve to the purposes of the study. All participants had normal or corrected-to-normal vision (50 and 25 subjects, respectively) and all of them reported to have no hearing problems, although no audiometric tests were performed to confirm this. Ages ranged between 19 and 50 years (average = 25.0 years; s.d. = 5.9 years). The experiments were undertaken with the understanding and written consent of each subject, following the Code of Ethics of the World Medical Association (Declaration of Helsinki), and were approved by the Ethics Committee of the Universidad Nacional de Quilmes. All study participants provided written informed consent, and received no payment for their time. The participants were recruited through advertisements distributed via group e-mails and announcement on Facebook community pages.

Participants were randomly assigned to two groups: subjects assigned to Group 1 performed the Experiment 1 in the anechoic chamber (*n* = 37) while subjects assigned to Group 2 performed it in the reverberant room (*n* = 38). In Experiment 2 subjects were interchanged between rooms: subjects from Group 1 performed the Experiment 2 in the reverberant room while subjects from Group 2 performed it in the anechoic chamber. For this reason, we refer to Group 1 as A → R, and to Group 2 as R → A.

### General procedure and experimental set-up

Each participant was received by the experimenter in the entrance hall (point B in Fig. 1) and brought into the reception room (C) where he/she received initial instructions on the task. The instructions were written in order to control variability in the understanding of the task by the subjects. At this point, and in order to induce familiarity with the target, the participant was able to touch and see an identical model of the visual targets employed in the task. Prior knowledge of the target could serve as a cue for visual distance, given the principle of size-distance invariance^58^. Then the participant was blindfolded in the reception room (C) and brought by the experimenter to the room assigned for Experiment 1 (D or E), where he/she was seated at the zero position. After the participant was located in the experimental chair, the experimenter left the room and the lights were turned off, remaining so until the completion of the experiment.

The red line in Fig. 1 indicates the path walked by the subjects since they were received until they were dismissed after completion of the whole experiment. The fact that both rooms were located facing each other, added to the similar location of the set-ups within each room, was fundamental for the participants of both groups to follow very similar paths. This fact allowed us to minimize the differences in proprioceptive information across conditions.

Before starting the experiment the participant was asked to remove the blindfold. In order to expose the subject to the acoustic characteristics of the room, a recording with the instructions of the experiment was played through a loudspeaker located 70 cm in front of the participant at a height of 1 m. The set-up also had a microphone in the room allowing real-time communication between the experimenter and the participant. Previous studies have shown that speech signals are effective stimuli in order to induce a spatial sense of the environment through audition^46, 50^.

Each target’s luminance was adjusted using as reference a value of 0.18 lux measured at a distance of 10 cm (ILT 1400-A portable radiometer, International Light Technologies Inc., Peabody, MA). This value, on the one hand, ensured that the targets were clearly distinguishable and, on the other, prevented illumination of any other surface in the rooms (e.g., walls, floor). Targets were also surrounded with black opaque fabric, in order to minimize illumination of such surfaces. In addition, to make sure that each room was perceived totally dark after eye’s adaptation, two experimenters performed, as if they were subjects, the complete procedure in both rooms. Finally, each participant was asked to report about any light source in the room besides the targets. None of the participants reported the presence of such undesired light leaks during the experiment.

Visual stimuli were acrylic squares of size 5 × 5 cm. Each target was illuminated by four LEDs mounted on its back that provided diffuse light. Stimuli were placed in front of the participant (0° azimuth) at a height of 1.50 m (approximately the height of the subject’s head, giving roughly 0° elevation). The experimental set-up consisted of a linear array of five visual stimuli, located at *D* = 2, 3, 4, 5 and 6 meters from the subject’s position (Fig. 1). Each stimulus was mounted on a metal support. To avoid visual obstruction between stimuli, servomotors were used to move the targets outside the participant’s line of sight. White noise (500 ms of duration) was presented through the loudspeaker before each trial, in order to mask servomotors noise, which could act as a cue for target distance. The masker also contributed to expose the subject to the acoustic characteristics of the room. The targets were lit only after they reached their final position and lasted on for 2 s. See Supplemental Figure S6 for detailed measurements of intensity levels for task instructions, masker and servomotors noise.

After each presentation, the participant was asked to express verbally the apparent distance to the visual target. Responses were recorded on a computer and manually transcribed into a data sheet by the experimenter, who remained outside the room until completion of the procedure (see Fig. 1). Each target was presented five times for each test distance (25 trials in total) in random order. Each participant made only one verbal response per trial and did not receive any information about the correctness of his/her responses. After completion of the VDP task, the subject was blindfolded and brought back to the reception room (C). There he/she filled a form with his/her estimation of the length, width and height of the room where the experiment was conducted. After completing this form, the subject was blindfolded again and brought into the other room assigned for Experiment 2, where the procedure (including the reproduction of the instructions through the loudspeaker) was identically repeated.

### Statistical methods

All data was analyzed considering a significance level of 5%. Visual distance perception curves obtained in the two rooms were compared by means of analysis of variance on the logarithm of the responses, with factors “target distance” (within-subjects) and “room” (between- or within-subjects, depending on the case) as fixed factors. Data from Experiment 1 was compared between groups, while data of Experiment 2 was compared to data from Experiment 1 within each group. In the case that sphericity was violated, reported *p*-values correspond to those obtained after applying the Greenhouse-Geiser correction (note that violations of sphericity can only occur for “target distance” and for the interaction between “target distance” and “room”, but not for “room”).

Individual room size data were compared between rooms by means of one-tailed, paired (when comparing within a group) or two-sample (when comparing between groups), *t*-tests, the null hypothesis being that responses in the reverberant room were *smaller than or equal to* responses in the anechoic chamber. The null hypothesis for these analyses was derived from previous evidence associating higher reverberation with larger perceived room size, as described in the Introduction. Data was log-transformed before the test, in order to achieve equality of variance across target distance. Given that the subtraction between two numbers in log-scale corresponds to the logarithm of their ratio in linear scale [i.e., log(*x*) − log(*y*) = log(*x*/*y*), (Eq. 1)], mean differences between room size (in log-scale) were transformed into mean “reverberant-to-anechoic ratios”, calculated as exp(mean difference reported by the *t*-test). This magnitude can be easily interpreted as its value is expected to be greater than one in case of rejection of the null hypothesis.

Post-analysis on the VDP curves were performed by means of one-tailed paired *t*-tests on the individual log-responses, the null hypothesis being the same as for the room size data. The null hypothesis in this case was derived from our prediction that target distance in the reverberant room will be perceived farther than in the anechoic chamber. Significance was controlled by means of the Holm-Bonferroni procedure^52^ for five comparisons: given that there were five target distances, five is the maximum number of possible comparisons between both rooms.

## Electronic supplementary material

Supplementary Information

## References

[1] Da Vinci, L. *Trattato Della Pittura Di Leonardo Da Vinci* (Langlois, Paris, 1651).

[2] Cutting, J. E. & Vishton, P. M. Perceiving layout and knowing distances: The integration, relative potency, and contextual use of different information about depth. In Epstein, W. & Rogers, S. J. (eds) *Handbook of Perception and Cognition*, Vol 5: Perception of Space and Motion, 69–117 (Academic Press, San Diego, CA, 1995).

[3] Proffitt, D. R. & Caudek, C. Depth perception and the perception of events. In Healy, A. F. & Proctor, R. W. (eds) *Handbook of Psychology, Vol. 4: Experimental Psychology*, 213–236 (Wiley, New York, 2002).

[4] Loomis, J. M. & Knapp, J. M. Visual perception of egocentric distance in real and virtual environments. *Virtual and Adaptive Environments* **11**, 21–46 (2003).

[5] Loomis, J. & Philbeck, J. W. Measuring perception with spatial updating and action. In Klatzky, R. L., MacWhinney, B. & Behrman, M. (eds) *Embodiment, Ego-space, and Action*, 1–43 (Psychology Press, New York, 2008).

[6] CreemRegehr SH, Kunz BR (2010). Perception and action. Wiley Interdisciplinary Reviews: Cognitive Science.

[7] Howard, I. P. *Perceiving in Depth**, Vol. 3: Other Mechanisms of Depth Perception* (Oxford Psychology Series, 2012).

[8] Semmlow JL, Heerema D (1979). The role of accommodative convergence at the limits of fusional vergence. Investigative Ophthalmology & Visual Science.

[9] Foley JM (1980). Binocular distance perception. Psychological Review.

[10] Ono H, Rogers BJ, Ohmi M, Ono ME (1988). Dynamic occlusion and motion parallax in depth perception. Perception.

[11] Qian N (1997). Binocular disparity and the perception of depth. Neuron.

[12] Bülthoff I, Bülthoff H, Sinha P (1998). Top-down influences on stereoscopic depth-perception. Nature Neuroscience.

[13] Loomis JM (2001). Looking down is looking up. Nature.

[14] Blake, R. & Sekuler, R. *Perception* (McGraw-Hill, New York, 2006), 5th edition.

[15] Sousa R, Brenner E, Smeets JBJ (2010). A new binocular cue for absolute distance: Disparity relative to the most distant structure. Vision Research.

[16] Thomson JA (1983). Is continuous visual monitoring necessary in visually guided locomotion?. Journal of Experimental Psychology: Human Perception and Performance.

[17] Elliott D (1986). Continuous visual information may be important after all: A failure to replicate Thomson (1983). Journal of Experimental Psychology: Human Perception and Performance.

[18] Loomis JM, Da Silva JA, Fujita N, Fukusima SS (1992). Visual space perception and visually directed action. Journal of Experimental Psychology: Human Perception and Performance.

[19] Fukusima SS, Loomis JM, Da Silva JA (1997). Visual perception of egocentric distance as assessed by triangulation. Journal of Experimental Psychology: Human Perception and Performance.

[20] Gogel WC (1961). Convergence as a cue to absolute distance. The Journal of Psychology.

[21] Philbeck JW, Loomis JM (1997). Comparison of two indicators of perceived egocentric distance under full-cue and reduced-cue conditions. Journal of Experimental Psychology: Human Perception and Performance.

[22] He ZJ, Wu B, Ooi TL, Yarbrough G, Wu J (2004). Judging egocentric distance on the ground: Occlusion and surface integration. Perception.

[23] Wu B, Ooi TL, He ZJ (2004). Perceiving distance accurately by a directional process of integrating ground information. Nature.

[24] Stefanucci JK, Proffitt DR, Banton T, Epstein W (2005). Distances appear different on hills. Perception & Psychophysics.

[25] Lappin JS, Shelton AL, Rieser JJ (2006). Environmental context influences visually perceived distance. Perception & Psychophysics.

[26] Witt JK, Stefanucci JK, Riener CR, Proffitt DR (2007). Seeing beyond the target: Environmental context affects distance perception. Perception.

[27] Iosa M, Fusco A, Morone G, Paolucci S (2012). Walking there: Environmental influence on walking-distance estimation. Behavioural Brain Research.

[28] Proffitt, D. R. An action-specific approach to spatial perception. In Klatzky, R. L., MacWhinney, B. & Behrman, M. (eds) *Embodiment, Ego-space, and Action*, 179–202 (Psychology Press, New York, 2008).

[29] Bingham GP, Pagano CC (1998). The necessity of a perception-action approach to definite distance perception: Monocular distance perception to guide reaching. Journal of Experimental Psychology: Human Perception and Performance.

[30] Coats RO, Pan JS, Bingham GP (2014). Perturbation of perceptual units reveals dominance hierarchy in cross calibration. Journal of Experimental Psychology: Human Perception and Performance.

[31] Rieser JJ, Pick HL, Ashmead DH, Garing AE (1995). Calibration of human locomotion and models of perceptual-motor organization. Journal of Experimental Psychology: Human Perception and Performance.

[32] Pan JS, Coats RO, Bingham GP (2014). Calibration is action specific but perturbation of perceptual units is not. Journal of Experimental Psychology: Human Perception and Performance.

[33] Bruggeman, H. & Warren, W. H. The direction of walking—but not throwing or kicking—is adapted by optic flow. *Psychological Science* (2010). 10.1177/0956797610372635 PMC3142708 20511390

[34] Bingham, G. P., Pan, J. S. & Mon-Williams, M. A. Calibration is both functional and anatomical. *Journal of Experimental Psychology: Human Perception and Performance* **40**, 61 (2014). 10.1037/a0033458 PMC3923309 23855525

[35] Ernst, M. O. & Bülthoff, H. H. Merging the senses into a robust percept. *Trends in Cognitive Sciences* **8**, 162–169 (2004). 10.1016/j.tics.2004.02.002 15050512

[36] Radeau M, Bertelson P (1974). The after-effects of ventriloquism. The Quarterly Journal of Experimental Psychology.

[37] Recanzone, G. H. Rapidly induced auditory plasticity: The ventriloquism aftereffect. *Proceedings of the National Academy of Sciences* **95**, 869–875 (1998). 10.1073/pnas.95.3.869 PMC33810 9448253

[38] Lewald J (2002). Rapid adaptation to auditory-visual spatial disparity. Learning & Memory.

[39] Alais D, Burr D (2004). The ventriloquist effect results from near-optimal bimodal integration. Current Biology.

[40] Hay JC, Pick HL, Ikeda K (1965). Visual capture produced by prism spectacles. Psychonomic Science.

[41] Pavani F, Spence C, Driver J (2000). Visual capture of touch: Out-of-the-body experiences with rubber gloves. Psychological Science.

[42] Ernst MO, Banks MS (2002). Humans integrate visual and haptic information in a statistically optimal fashion. Nature.

[43] Calcagno ER, Abregú EL, Eguía MC, Vergara R (2012). The role of vision in auditory distance perception. Perception.

[44] Gajewski DA, Philbeck JW, Wirtz PW, Chichka D (2014). Angular declination and the dynamic perception of egocentric distance. Journal of Experimental Psychology: Human Perception and Performance.

[45] Gajewski DA, Wallin CP, Philbeck JW (2014). Gaze behavior and the perception of egocentric distance. Journal of Vision.

[46] Kolarik AJ, Pardhan S, Cirstea S, Moore BC (2013). Using acoustic information to perceive room size: Effects of blindness, room reverberation time, and stimulus. Perception.

[47] Sandvad J (1999). Auditory perception of reverberant surroundings. The Journal of the Acoustical Society of America.

[48] McGrath, R., Waldmann, T. & Fernström, M. Listening to rooms and objects. In *Audio Engineering Society Conference: 16th International Conference: Spatial Sound Reproduction* (Audio Engineering Society, 1999).

[49] Mershon DH, Ballenger WL, Little AD, McMurtry PL, Buchanan JL (1989). Effects of room reflectance and background noise on perceived auditory distance. Perception.

[50] Hameed, S., Pakarinen, J., Valde, K. & Pulkki, V. Psychoacoustic cues in room size perception. In *Audio Engineering Society Convention 116* (Audio Engineering Society, 2004).

[51] Cabrera, D., Jeong, D., Kwak, H. J., Kim, J. Y. & Duckjin-gu, J. Auditory room size perception for modeled and measured rooms. In *Internoise, the 2005 Congress and Exposition on Noise Control Engineering* (Rio de Janeiro, Brazil, 2005).

[52] Holm, S. A simple sequentially rejective multiple test procedure. *Scandinavian Journal of Statistics, ***6**, 65–70 (1979).

[53] Loomis JM, Klatzky RL, Philbeck JW, Golledge RG (1998). Assessing auditory distance perception using perceptually directed action. Attention, Perception, & Psychophysics.

[54] King AJ (2009). Visual influences on auditory spatial learning. Philosophical Transactions of the Royal Society of London B: Biological Sciences.

[55] Zahorik P (2001). Estimating sound source distance with and without vision. Optometry & Vision Science.

[56] Velis, A. G., Giuliano, H. G. & Méndez, A. M. The anechoic chamber at the Laboratorio de Acústica y Luminotecnia CIC. *Applied Acoustics* **44**, 79–94 (1995).

[57] Giuliano, H. G., Velis, A. G. & Méndez, A. M. The reverberation chamber at the Laboratorio de Acústica y Luminotecnia of the Comisión de Investigaciones Científicas. *Applied Acoustics* **49**, 71–83 (1996).

[58] Welch, R. B. *Perceptual Modification: Adapting to Altered Sensory Environments* (Elsevier, 2003).

