# Supplementary material for: Auditory environmental context affects visual distance perception
Source: Sci Rep. 2017 Aug 3;7:7189. doi: 10.1038/s41598-017-06495-3 (PMC5543138; doi:10.1038/s41598-017-06495-3)

Auditory environmental context affects visual distance perception

Pablo Esteban Etchemendy[1], Ezequiel Abregú[1], Esteban Ramón Calcagno[1], Manuel Camilo Eguía[1], Nilda Vechiatti[2], Federico Iasi[2] and Ramiro Oscar Vergara[1,*]

[1] Laboratorio de Acústica y Percepción Sonora, Escuela Universitaria de Artes, CONICET, Universidad Nacional de Quilmes, B1876BXD, Bernal, Argentina.

[2] Laboratorio de Acústica y Luminotecnia. Comisión de Investigaciones Científicas de la Pcia. de Bs. As. Cno. Centenario e/ 505 y 508, M. B. Gonnet, Bs. As., Argentina.

[*] Correspondence concerning this article should be addressed to Ramiro Vergara: ramirovergara@lapso.org

This document contains the list of supplemental figures and tables

Supplemental Table S1: Reverberation time measured in both testing environments at the listener seat, with the experimental set-up present. The reverberation time is defined as the time it takes for the sound level in the room to decrease by 60 dB after a continuous sound source has been shut off (Springer Handbook of Acoustics, Rossing, ed., 2007; see Sec. 9.3.1). Values were measured for third-octave frequency bands in the range 50 Hz-10 kHz (Brüel & Kjaer 2250 sound level meter) through the parameter T_{20}, defined as three times the time required to observe a 20-dB decay between 5 and 25 dB below the initial level of the sound source (ISO standard 3382-1:2009: "Measurement of room acoustic parameters - Part 1: Performance spaces"). In the anechoic chamber, the 50- and 63-Hz bands could not be characterized due to poor sound generation in that frequency regions. Mean values for the six bands in the range 400-1250 Hz (following ISO standard 3382-1:2009) are reported for each environment (bottom row). Despite having no physical significance, they serve the purpose of summarizing in a single value the room response across bands.

**Band [Hz]**	**T_{20} [s]**	
	**Reverberant Room**	**Anechoic Chamber**
50	7.95	-
63	7.58	-
80	5.92	0.42
100	5.58	0.24
125	5.18	0.23
160	5.39	0.24
200	4.43	0.14
250	4.64	0.10
315	4.57	0.17
400	4.17	0.10
500	3.78	0.12
630	3.78	0.12
800	3.70	0.08
1000	3.81	0.08
1250	3.89	0.09
1600	3.78	0.09
2000	3.33	0.11
2500	2.95	0.09
3150	2.58	0.09
4000	2.15	0.09
5000	1.75	0.10
6300	1.43	0.09
8000	1.03	0.10
10000	0.80	0.10
**Mean across 400-1250 Hz**	**3.86**	**0.10**

Supplemental Figure S2: Individual maximum perceived distance (MPD) vs. perceived room volume for both groups (rows) in Experiments 1 and 2 (columns). For each condition, we report (see inset) the correlation coefficient between both magnitudes. All groups showed a significant positive correlation (indicated by asterisks, significance level 5%) with exception of Group 1 in the reverberant room (Exp. 2). The red line indicates the best linear fit for the data.

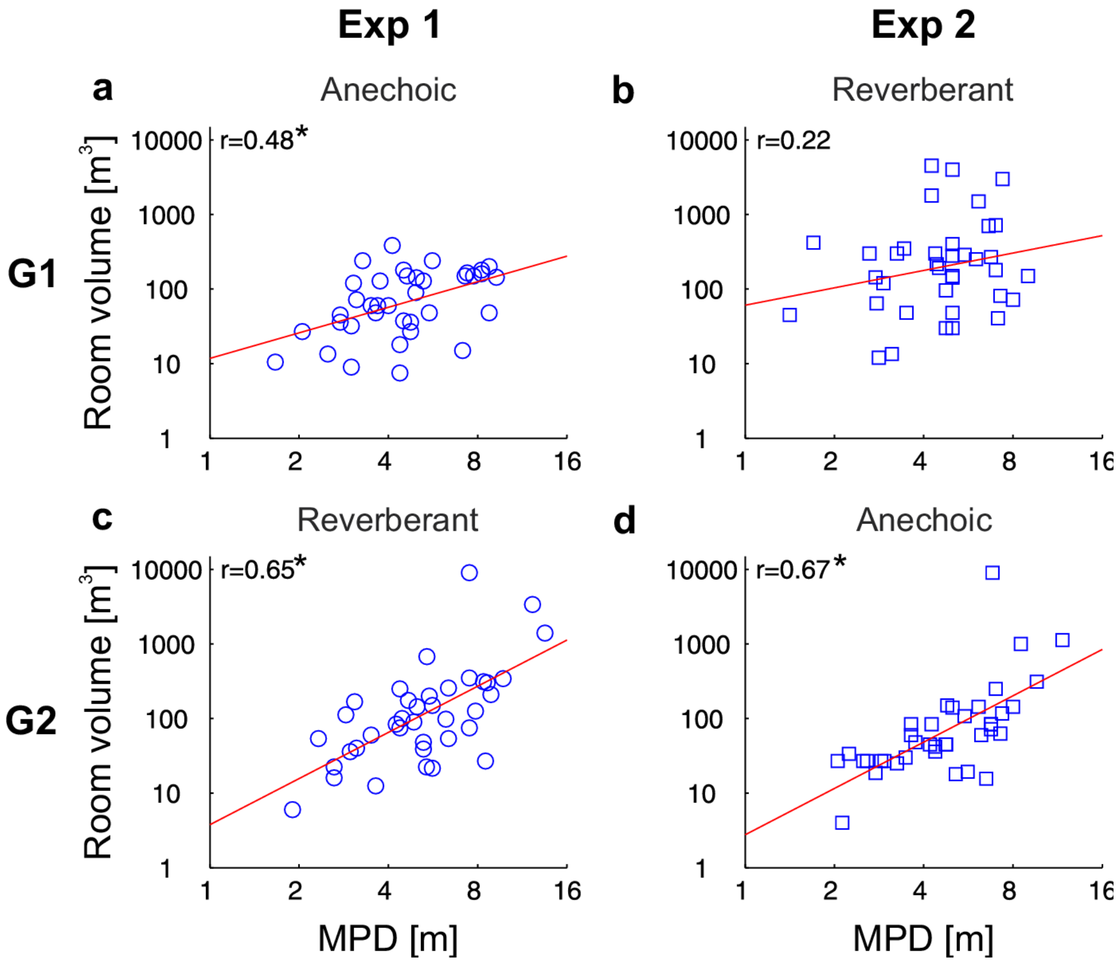

Supplemental Figure S3: Individual slopes relating target distance with distance responses plotted against perceived room volume for both groups (rows) in Experiments 1 and 2 (columns). For each condition, we report (see inset) the correlation coefficient between both magnitudes. All groups showed a significant positive correlation (significance level 5%) with exception of Group 1 in the reverberant room (Exp. 2). The red line indicates the best linear fit for the data.

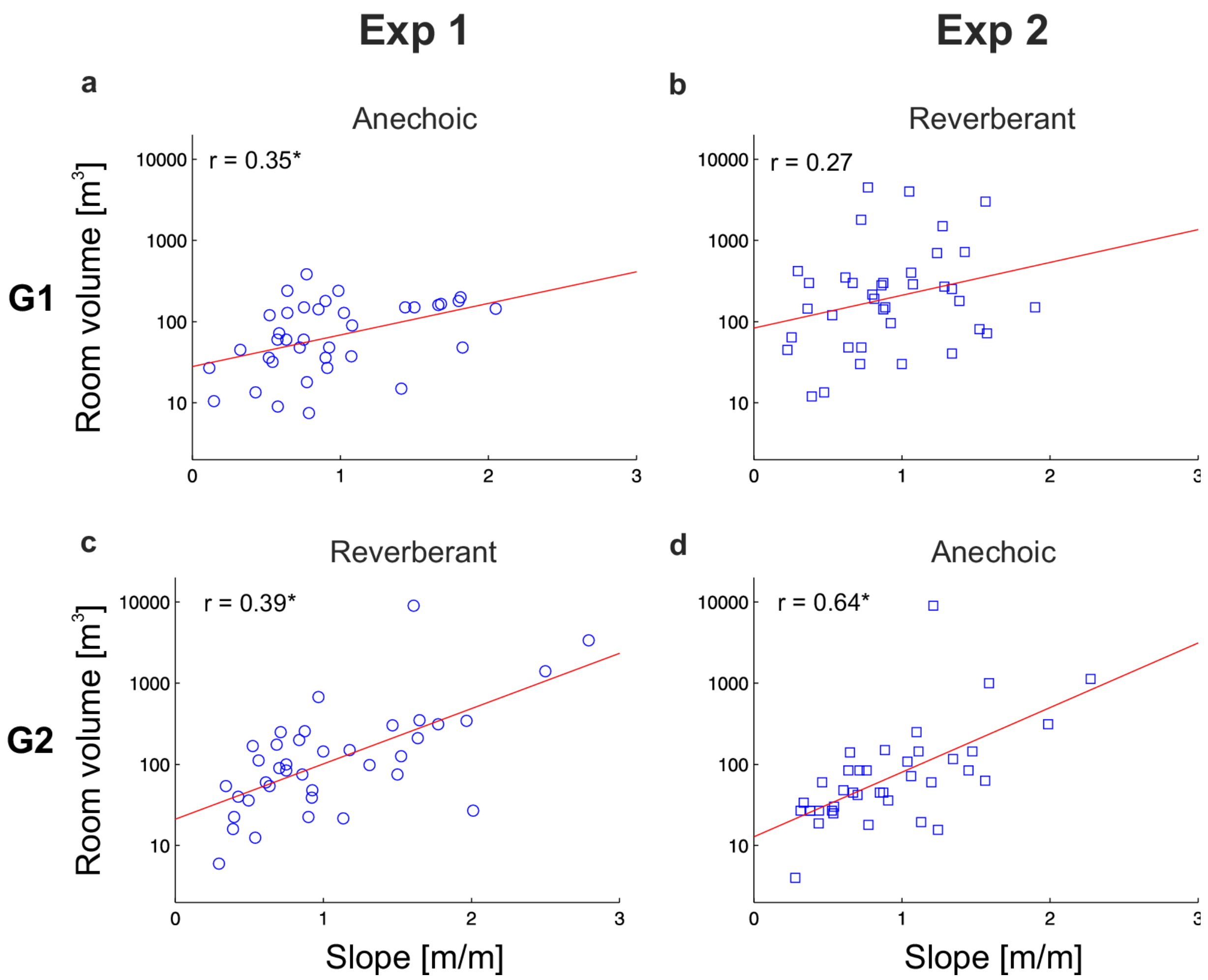

Supplemental Table S4. Mean values (+/- SEM) across subjects for the perceived length, width, and height for each experimental condition.

	**Exp. 1**	**Exp. 2**
**Group 1 (A→R)**	Length: 6.55 +/- 0.47 m<br>Width: 3.72 +/- 0.26 m<br>Height: 3.37 +/- 0.16 m	Length: 9.82 +/- 0.99 m<br>Width: 6.73 +/- 0.89 m<br>Height: 4.85 +/- 0.44 m
**Group 2 (R→A)**	Length: 8.03 +/- 0.85 m<br>Width: 5.64 +/- 0.83 m<br>Height: 4.12 +/- 0.41 m	Length: 6.89 +/- 0.79 m<br>Width: 4.56 +/- 0.80 m<br>Height: 3.47 +/- 0.29 m

Supplemental Table S5. Incompatibility between room size and maximum perceived distance (MPD). For a given condition, we defined the *incompatibility* index (II) as the percentage of subjects whose responses fulfilled the inequality: MPD ≥ Perceived Room Length. We first computed the II for the four possible combinations between Group and Room (four upper rows). The II applied to this cases gives the baseline value for the percentage of subjects reporting a room smaller than the MPD within the same room. The mean baseline value was 14.7% with 95% CI = +/-3.4%, a relatively low value. We next computed the II for the data of each group across rooms, comparing the perceived length in Experiment 2 with the MPD in Experiment 1 (two lower rows). In this way, we checked if the subjects compressed their VDP response in Experiment 2 (with respect to Experiment 1) due to changes in perceived room size across rooms. Across experiments, subjects of Group 2 (R→A) showed an II almost two times the baseline value, consistent with a reduction of the perceived space in the reverberant room compared to the anechoic chamber. On the other hand, subjects of Group 1 (A→R) showed an II across experiments almost one half the baseline value, consistent with an expansion of the perceived space when entering the reverberant room after leaving the anechoic chamber.

**Condition**	**Incompatibility index**
Group 1 in A [within Exp. 1]	18.9%
Group 2 in A [within Exp. 1]	15.8%
Group 1 in R [within Exp. 2]	10.8%
Group 2 in R [within Exp. 2]	13.2%
Group 1 across rooms: MPD(A) ≥ Length(R)	8.1%
Group 2 across rooms: MPD(R) ≥ Length(A)	28.9%

Abbreviations: R, reverberant room; A, anechoic chamber.

Supplemental Figure S6: Sound pressure level measured in both testing environments at the listener's seat for the background noise, servomotors noise, masker and task instructions. Spectra values (Z-weighted Equivalent Continuous Sound Pressure Level, or LZEq) were measured for third-octave frequency bands in the range from 50 Hz to 10 kHz (Brüel & Kjaer 2250 sound level meter). The masker was adjusted to ensure masking of the servomotors noise. Servomotors were measured when moving in both directions (up and down), four times each in each direction. For each direction we plot the average across all motors and repetitions. Global-value measurements (A-weighted Equivalent Continuous Sound Pressure Level, or LAeq) in the reverberant room were: noise background, 23 dB; servomotors noise (up and down avg.), 37 dB; masking noise, 57 dB; and instructions, 65 dB. Measurements in the anechoic chamber were: noise background, 18 dB; servomotors noise (up and down avg.), 31 dB; masking noise, 65 dB; and task instructions, 68 dB.

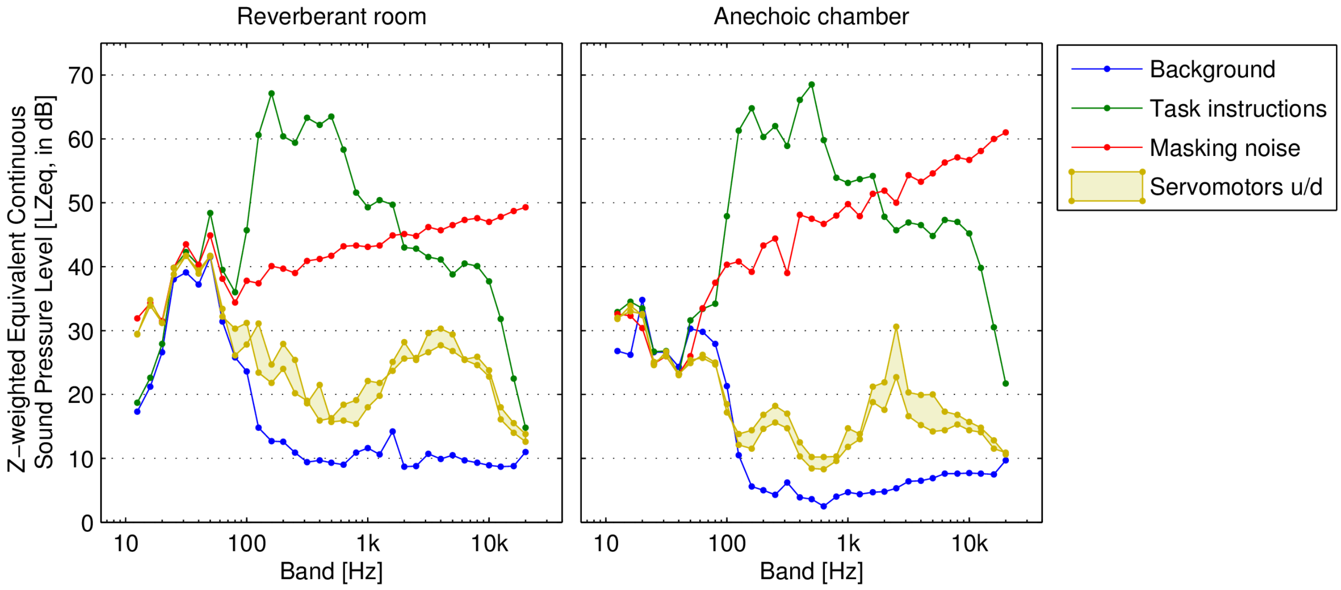

Supplement: Supplementary file 1 — Supplementary Information [file 41598_2017_6495_MOESM1_ESM.pdf]
